# Structure of *Toxoplasma gondii* fructose-1,6-bisphosphate aldolase

**DOI:** 10.1107/S2053230X14017087

**Published:** 2014-08-29

**Authors:** Lauren E. Boucher, Jürgen Bosch

**Affiliations:** a Department of Biochemistry and Molecular Biology, Johns Hopkins Bloomberg School of Public Health, 615 North Wolfe Street, Baltimore, MD 21205, USA; b Johns Hopkins Malaria Research Institute, Johns Hopkins Bloomberg School of Public Health, 615 North Wolfe Street, Baltimore, MD 21205, USA

**Keywords:** *Toxoplasma*, aldolase, glideosome, invasion, MIC2, F16BP

## Abstract

The apicomplexan parasite *Toxoplasma gondii* must invade host cells to continue its lifecycle. It invades different cell types using an actomyosin motor that is connected to extracellular adhesins *via* the bridging protein fructose-1,6-­bisphosphate aldolase. During invasion, aldolase serves in the role of a structural bridging protein, as opposed to its normal enzymatic role in the glycolysis pathway. Crystal structures of the homologous *Plasmodium falciparum* fructose-1,6-bisphosphate aldolase have been described previously. Here, *T. gondii* fructose-1,6-bisphosphate aldolase has been crystallized in space group *P*22_1_2_1_, with the biologically relevant tetramer in the asymmetric unit, and the structure has been determined *via* molecular replacement to a resolution of 2.0 Å. An analysis of the quality of the model and of the differences between the four chains in the asymmetric unit and a comparison between the *T. gondii* and *P. falciparum* aldolase structures is presented.

## Abstract 2

The structure of *T. gondii* fructose-1,6-bisphosphate aldolase, a glycolytic enzyme and structural component of the invasion machinery, was determined to a resolution of 2.0 Å.

## 1. Introduction   

Toxoplasmosis is a disease caused by the apicomplexan parasite *Toxoplasma gondii* and is a leading cause of death *via* food-borne illness (Jones & Dubey, 2012 ▶; Jones *et al.*, 2014 ▶). Seroprevalence estimates range from less than 10% in China to 10–20% in the United States, 20–40% in Australia and parts of Africa, 40–60% in European and South American countries and as high as 77 and 83% in Brazil and Madagascar, respectively (Sepulveda-Arias *et al.*, 2014 ▶). The majority of people infected with *T. gondii* will never experience symptoms of the disease; however, those who are immunocompromised owing to HIV/AIDS, chemotherapy or taking immuno­suppressants, the elderly and pregnant women are at serious risk if they become infected (Jones *et al.*, 2014 ▶).

*T. gondii* is an apicomplexan parasite belonging to the same family as the malaria-causing parasite *Plasmodium falciparum*. Both parasites are obligate intracellular parasites that must invade host cells to survive. *T. gondii* has often been used to study pathways and mechanisms common to both parasites owing to the large toolbox for genetic manipulation available in *T. gondii*. One pathway that both apicomplexan parasites share and utilize is the invasion pathway. To invade a host cell, the parasites must attach to the surface *via* extracellular adhesins and employ an actomyosin-based motor to invade the cell. Many components of this invasion machinery, also known as the glideosome, are common to both parasites (Kappe *et al.*, 1999 ▶, 2004 ▶; Bergman *et al.*, 2003 ▶). In order to transmit the mechanochemical force generated by the motor to the extracellular adhesins in contact with the host cell, a bridging protein is required. The parasites use the tetrameric fructose-1,6-bisphosphate aldolase to make this connection (Jewett & Sibley, 2003 ▶; Bosch *et al.*, 2007 ▶).

Aldolase is a glycolytic enzyme; however, it is recruited to the invasion machinery to serve in a structural capacity. Previous work has identified that the C-terminal cytoplasmic tails of key parasite adhesins bind to the aldolase enzyme active site (Bosch *et al.*, 2007 ▶) and actin binds at another interface (St-Jean *et al.*, 2007 ▶). These two interactions connect the intracellular motor to the extracellular adhesins. Here, we present the 2.0 Å resolution structure of fructose-1,6-bisphosphate aldolase from *T. gondii* (TgAldolase), providing structural detail regarding a key component of the *Toxoplasma* invasion machinery and details of the adhesin-binding pocket.

## 2. Materials and methods   

### 2.1. Macromolecule production   

The TgAldolase clone was provided by the Seattle Structural Genomics Center for Infectious Disease (SSGCID) and transformed into *Escherichia coli* BL21 (DE3) cells. SSGCID uses the multi-construct length approach when cloning, typically generating multiple clones of different lengths. The multiple constructs are then expressed and tested for solubility and yield. We requested this clone, which lacks seven N-terminal residues and 23 C-terminal residues, since it was noted as having a high expression level with a solubility of at least 26.65 mg ml^−1^. Details of the construct used, including the expected protein sequence, molecular weight and extinction coefficients, are presented in Table 1 ▶.

A single colony was used to inoculate 2 l Terrific Broth (TB) medium containing 50 µg ml^−1^ ampicillin. Cultures were grown at 310 K with shaking at 250 rev min^−1^. Expression was induced at an OD_600_ of 3.0 with 0.5 m*M* isopropyl β-d-1-thiogalactopyranoside (IPTG) and continued for 18 h at 293 K. Cells were harvested *via* centrifugation at 1370*g* for 30 min at 277 K, resuspended in lysis buffer [50 m*M* Tris pH 8.0, 500 m*M* NaCl, 20 m*M* imidazole, 5%(*v*/*v*) glycerol] and lysed using an Emulsiflex C5 cell disruptor at 100 MPa. Benzonase (Sigma) and cOmplete protease-inhibitor tablets (Roche) were added to the lysate, which was spun down at 38 725*g* for 45 min at 277 K. The supernatant was then batch-bound with 4 ml Co-TALON (Clontech) resin equilibrated in lysis buffer for 45 min at 277 K. The resin was washed with 50 ml lysis buffer and the protein was eluted with 35 m*M* Tris pH 8.0, 350 m*M* NaCl, 314 m*M* imidazole. The protein yield was approximately 1.3 mg per gram of cell paste. The protein was then dialyzed overnight at 277 K into 50 m*M* Tris pH 8.0, 50 m*M* NaCl, 1 m*M* dithiothreitol (DTT), 2%(*v*/*v*) glycerol and passed over a HiLoad Superdex 200 (GE Healthcare) column. Fractions containing TgAldolase were combined and concentrated to 20 mg ml^−1^ as determined from the *A*_280_ using a NanoDrop 1000 (Thermo Scientific) and an extinction coefficient of 23 170 *M* ^−1^ cm^−1^, as calculated for the reduced protein using *ProtParam* (Gasteiger *et al.*, 2005 ▶). The final protein yield after purification was approximately 1.2 mg per gram of cell paste. The protein was then flash-cooled in liquid nitrogen and stored at 193 K before crystallization.

### 2.2. Crystallization   

Initial crystallization screens for TgAldolase were performed using MORPHEUS (Gorrec, 2009 ▶), Wizard (Emerald Bio), NeXtal Classic (QIAGEN) and Crystal Screens 1 and 2 (Hampton Research) in 96-well three-drop Intelli-Plates using a Mosquito for rapid setup. The top two hits from the MORPHEUS screen (A7 and G7) consisted of MOPS/HEPES–Na pH 7.5, glycerol, PEG 4000 and either carboxylic acids or divalent ions. Further optimization trays were set up in 24-well plates to optimize the pH, precipitant and salt concentration, as well as the carboxylic acid and divalent ion composition. Optimization plates were seeded using crushed crystals from previous screens. During the optimization process it was determined that the carboxylic acid conditions produced better crystals than the divalent ion conditions, and a mixture of divalent ions and carboxylic acids did not improve the crystal quality. Room-temperature setup and incubation at 293 K produced crystals that grew within 24 h; however, they only diffracted to ∼6 Å resolution. Setup on ice and incubation at 277 K produced large (>1 mm) crystals that diffracted to ∼1.7 Å resolution when properly cryoprotected. Crystallization details are presented in Table 2 ▶.

### 2.3. Data collection and processing   

Crystals were soaked in cryosolution, which was a mixture of reservoir solution and glycerol with a final glycerol concentration of 35%(*v*/*v*), and flash-cooled in liquid nitrogen. Diffraction data were collected remotely on beamline 7-1 at the Stanford Synchrotron Radiation Lightsource (SSRL). Data were processed and scaled using *XDSGUI* as a front end to *XDS*/*XSCALE* (Kabsch, 2010 ▶). Processing statistics are presented in Table 3 ▶.

### 2.4. Structure solution and refinement   

The structure was solved by molecular replacement using *Phaser* (McCoy *et al.*, 2007 ▶) in the *CCP*4 suite (Winn *et al.*, 2007 ▶) with a homology model of TgAldolase generated by *I-TASSER* (Zhang, 2008 ▶; Roy *et al.*, 2010 ▶) with a user-provided template, PDB entry 2pc4 (the *P. falciparum* homolog; PfAldolase; Bosch *et al.*, 2007 ▶). The sequence identity between PfAldolase and TgAldolase is 69% and the 2pc4 structure covered 93% of the primary sequence of TgAldolase. Rounds of automatic model building with *Buccaneer* (Cowtan, 2006 ▶, 2008 ▶) and density modification using *Parrot* (Zhang *et al.*, 1997 ▶) and *phenix.phase_and_build* (Adams *et al.*, 2010 ▶) to extend the initial models were used to build a starting model. Further refinement and loop building were performed using *phenix.refine* (Adams *et al.*, 2010 ▶) and *Coot* (Emsley *et al.*, 2010 ▶). During initial rounds of refinements, NCS restraints were used in addition to secondary-structure restraints and optimization of X-ray and geometry weights was performed. Refinement with NCS restraints was discontinued during later stages of refinement owing to large differences between chains *A* and *D* and the problem of shifting residues in the other chains. TLS groups were automatically determined *via* *PHENIX* (Adams *et al.*, 2010 ▶) and used in the second half of the refinement process. The quality of the final structure was validated *via* *MolProbity* (Chen *et al.*, 2010 ▶). Modeling and refinement statistics are presented in Table 4 ▶.

## 3. Results and discussion   

TgAldolase was crystallized in the presence of glycerol and PEG 4000 at 277 K, producing crystals that were cryoprotected with a cryo­solution containing reservoir and glycerol at a final concentration of 35%(*v*/*v*) before flash-cooling in liquid nitrogen and data collection at the synchrotron. Crystal growth at 277 K and the appropriate cryosolution improved the resolution of diffraction by 4 Å when compared with the crystals incubated at 293 K and similarly treated with cryosolution. Details of the crystallization method are given in Table 2 ▶. This improvement in crystal quality and appropriate cryosolution enabled us to solve the structure. Diffraction data were collected, processed and scaled to a resolution of 2 Å with 96.72% completeness over the entire data set and an average *I*/σ(*I*) of 15.36. Details of the data-collection and processing statistics can be found in Table 3 ▶. Fine-sliced, high-resolution reflection statistics produced by *XDS*/*XSCALE* can be found in Supplementary Table S1^1^.

**Figure 1:**
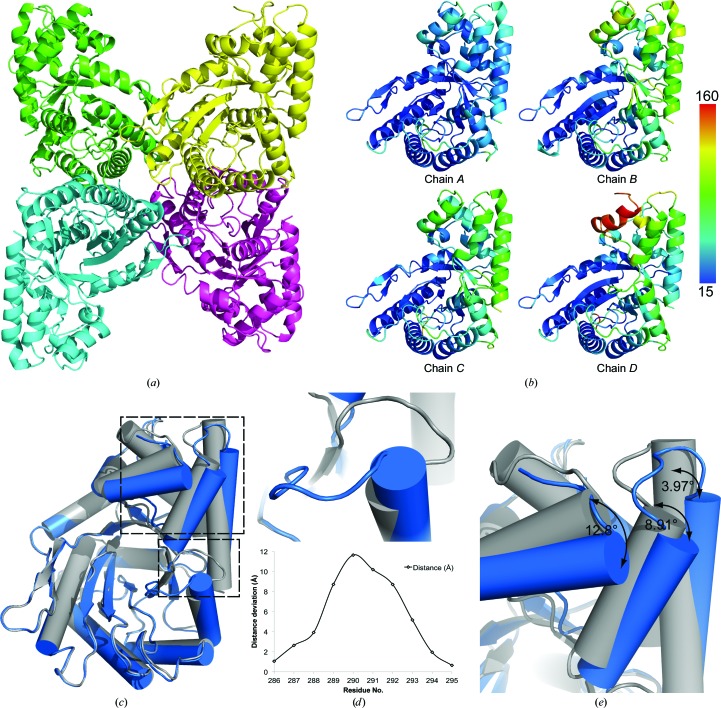
(*a*) Cartoon representation of the TgAldolase tetramer with individually colored monomers. (*b*) Aligned chains colored by *B* factor in units of Å^2^. (*c*) Chain *D* (blue) aligned with chain *A* (gray) over the residue range 176–198 in the same orientation as the yellow chain in the tetramer above. (*d*) Depiction of the loop shift in chain *D* compared with chain *A*, with a plot representing the inter-residue C^α^-atom deviation between residues 286 and 295. (*e*) Measurement of the degree of shift of α-helices 2, 11 and 12 between chains *D* and *A*. Figures were generated using *PyMOL* (Schrödinger).

TgAldolase crystallized in space group *P*22_1_2_1_, with four copies in the asymmetric unit forming the biologically relevant tetramer, with unit-cell parameters *a* = 92.26, *b* = 134.46, *c* = 162.43 Å, α = β = γ = 90°. The solvent content of the cell is 61.0% with a Matthews coefficient of 3.15 Å^3^ Da^−1^ as calculated *via* *phenix.xtriage* (Matthews, 1968 ▶; Adams *et al.*, 2010 ▶). As we did not cleave the purification tag, we expected a full-length structure containing 355 amino acids; however, the first 14 amino acids, which include the purification tag and a portion of the 3C protease cleavage site, were unresolved in the structure. The C-terminus is completely modeled in all four chains. The structure was determined to a resolution of 2.0 Å (Fig. 1 ▶ *a*) and refined with an *R*_work_ of 19.40% and *R*_free_ of 24.42%. Details of the refinement can be found in Table 4 ▶. The final set of coordinates and the corresponding structure factors have been deposited as Protein Data Bank entry 4tu1.

### 3.1. Comparison of individual chains in the asymmetric unit   

While the four chains are identical in sequence, barring missing amino-terminal residues, there are structural and quality differences amongst the chains. A graphical representation of the *B* factors is shown in Fig. 1 ▶(*b*), indicating that overall chain *A* has the lowest *B* factors, while chains *B*, *C* and *D* have elevated *B* factors in some areas. Chain *D* has high *B* factors in α-helix 2. The secondary structure is annotated according to *ENDscript* 2.0 (http://endscript.ibcp.fr; Gouet *et al.*, 2003 ▶; Supplementary Fig. S1). Furthermore, alignment of chains *B* and *C* with chain *A* using the *MUSTANG* server (Konagurthu *et al.*, 2010 ▶) gave a C^α^ root-mean-squared deviation (r.m.s.d.) of 0.41 and 0.28 Å over 340 residues, respectively, while chain *D* deviates by 1.63 Å over 333 residues.

Looking more closely at the differences in chain *D*, we find that α-helix 2 and the following unstructured stretch (residues 52–66) are poorly ordered, with little electron density to define the side chains and high *B* factors (Fig. 1 ▶ *b*). Density for residues 67–72 is completely absent, while it is well defined in the other three chains. Chain *D* was aligned with chain *A* using *Coot* and *LSQ superpose* (Emsley *et al.*, 2010 ▶) over the residue range 176–198. By comparing the aligned chains, we note several differences, including helix and loop shifts (Fig. 1 ▶ *c*). Three helices in chain *D* are tilted with respect to those in chain *A*: α-helix 2, α-helix 11 and α-helix 12 are tilted by 12.8, 8.91 and 3.97°, respectively. The tilt of α-helix 2 may be a result of the poorly resolved electron density that did not allow precise modeling of this helix, despite attempts to refine this region with secondary restraints, NCS averaging and rigid-body refinement to improve the modeling of this region.

**Figure 2:**
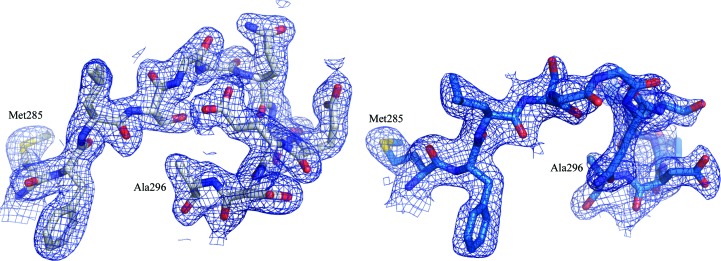
2*F*_o_ − *F*_c_ electron-density map (blue mesh) contoured at 1σ around residues 285–296 of chain *A* (left, gray) and chain *D* (right, blue), highlighting the different conformations of the loop connecting β-strand 10 to α-helix 10.

Additionally, a loop (residues 286–295) near the active site, connecting β-strand 10 to α-helix 10, is in a different orientation, with an up to 11.64 Å inter-C^α^-atom deviation between chains *A* and *D*; the inter-residue distances are plotted in Fig. 1 ▶(*d*). In Fig. 2 ▶, we show the 2*F*_o_ − *F*_c_ electron density for this stretch of residues at a contour level of 1σ, showing clearly defined density about the backbone and side chains, indicating that this is a true conformational difference in the loop and not an artifact caused by poorly resolved density in this region or owing to crystal contacts, as this region is not involved in packing. The importance of these shifts and alternate conformations will be discussed in comparison to *P. falciparum* aldolase.

### 3.2. Comparison of *T. gondii* and *P. falciparum* aldolase   

**Figure 3:**
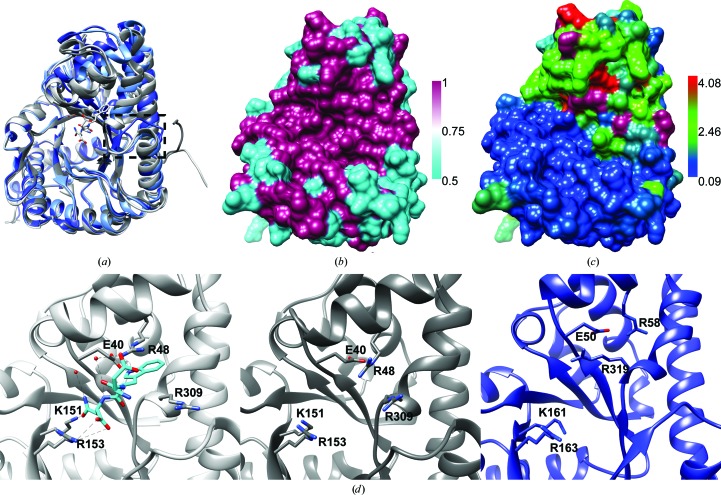
(*a*) Alignment *via* *Chimera* *MatchMaker* of PfAldolase (PDB entry 1a5c, chain *A*), PfAldolase bound to TRAP (PDB entry 2pc4, chain *D*) and TgAldolase (PDB entry 4tu1, chains *A* and *D*) represented as cartoons. The TRAP peptide shown in stick representation with two coordinating waters is present in the active site, which doubles as the adhesin tail binding pocket. A box highlights the dual conformation of the Met285–Ala296 loop in the TgAldolase chain *A* and *D* and PfAldolase structures. (*b*) Sequence conservation between TgAldolase and PfAldolase mapped on a surface representation of TgAldolase, with magenta indicating identical residues and cyan representing differences. (*c*) Surface representation of TgAldolase chain *A* colored according to residue r.m.s.d. (Å) from the reference structure PfAldolase (PDB entry 1a5c, chain *A*). (*d*) Three-panel enlargement of the adhesin-binding site of (left) TRAP bound to PfAldolase (PDB entry 2pc4), (middle) apo PfAldolase (PDB entry 1a5c) and (right) TgAldolase chain *A*, with residues important for TRAP binding highlighted as sticks.

As previously mentioned, the *T. gondii* system is often used to study that of the closely related parasite *P. falciparum*, which causes malaria. The structure of PfAldolase has previously been determined in an apo (Kim *et al.*, 1998 ▶) and a thrombospondin-related anonymous protein (TRAP)-bound form (Bosch *et al.*, 2007 ▶). Here, we compare the *T. gondii* and *P. falciparum* structures. An alignment and superposition of four aldolase chains, apo PfAldolase (PDB entry 1a5c, chain *A*), PfAldolase with TRAP bound (PDB entry 2pc4, chain *D*) and TgAldolase (PDB entry 4tu1, chains *A* and *D*), was performed using the *MatchMaker* tool in *Chimera* and is shown in Fig. 3 ▶(*a*). The C^α^ r.m.s.d. (calculated with 2 Å cutoff between residues) of the TRAP-bound PfAldolase chain from the apo PfAldolase structure was 0.547 Å (337 residues). The r.m.s.d. of TgAldolase chains *A* and *D* from the reference apo PfAldolase structure were 0.730 Å (222 residues) and 0.726 Å (221 residues), respectively. The overall C^α^ r.m.s.d.s calculated using the *MUSTANG* server (Konagurthu *et al.*, 2010 ▶) were 0.54, 2.44 and 2.16 Å for TRAP-bound PfAldolase, TgAldolase chain *A* and TgAldolase chain *D*, respectively. As mentioned previously, the sequence identity between *T. gondii* and *P. falciparum* aldolase is 69% and the residue conservation is mapped onto a surface representation of TgAldolase in Fig. 3 ▶(*b*).

When looking at the plotted sequence identity, we observe that the active site and adhesin-binding pocket are highly conserved (Fig. 3 ▶ *b*). However, upon inspection of the alignment of the different aldolase chains (Fig. 3 ▶ *a*) we noted that the structures do not align well in portions of the adhesin-binding site, so we next investigated the residue-by-residue r.m.s.d. between PfAldolase and TgAldolase. Using *Chimera* and *Match->Align*, we colored the surface of TgAldolase according to the deviation of each residue from the aligned residue in PfAldolase (Fig. 3 ▶ *c*). The lower half of the molecule in the orientation shown did diverge from the sequence of PfAldolase; however, this region, which makes contacts with the other chains to form the tetramer, has a low r.m.s.d. (∼0.1 Å) from the PfAldolase structure, as depicted by its blue coloring. Unlike the divergent in sequence, yet structurally similar, lower half of the molecule, the upper half has higher sequence identity yet the r.m.s.d. of the residues of the *T. gondii* and *P. falciparum* aldolase is larger.

A closer view of the adhesin-binding sites of PfAldolase and TgAldolase is presented in Fig. 3 ▶(*d*). The left panel highlights the residues important for adhesin binding in the TRAP-bound PfAldolase structure (PDB entry 2pc4). The middle and right panels show the apo PfAldolase and apo TgAldolase structures, respectively. We see that Lys151 and Arg153 of PfAldolase, which are responsible for interacting with the asparagine of the TRAP peptide (DWN), align well with the corresponding residues, Lys161 and Arg163, of TgAldolase. Two arginines, Arg48 and Arg309, of PfAldolase are important for binding of the TRAP peptide in PfAldolase. Arg309 moves so that the tryptophan of the TRAP peptide can insert between Arg48 and Arg309, which anchors the peptide in the binding site. It has been shown previously that this tryptophan is essential for the binding of adhesins to PfAldolase (Ménard, 2001 ▶; Buscaglia *et al.*, 2006 ▶). 

When we look at the orientation of the arginine residues in the TgAldolase structure, we find that the corresponding Arg58 and Arg319 are shifted or in alternate conformations. Arg58 resides in α-helix 2 of TgAldolase and we find that this helix is shifted away from the binding site compared with the position in the PfAldolase structures. Arg319 belongs to α-helix 11, which also deviates from the PfAldolase helix 11 position; however, this arginine is in an alternate conformation and completely blocks the adhesin-binding site where the key tryptophan docks. In order for adhesins to bind to TgAldolase, this arginine would need to rotate out by 90° to allow the tryptophan to insert between Arg319 and Arg58. The positioning of this helix 11 can be affected by the position of a loop just outside the active site. This loop, residues 286–295, was previously described in Fig. 2 ▶ to be in conformations that differed between chains *A* and *D*. The conformation of the loop connecting β-strand 10 to α-helix 10 in chain *A* does not align with the PfAldolase structure; however, the conformation in chain *D* aligns well. The alternate conformation seems to allow α-helices 2 and 11 to shift, as noted in Fig. 1 ▶(*e*), to a position that deviates less from the PfAldolase structures (Fig. 3 ▶ *a*). This flexibility indicates that TgAldolase may be able to adopt a conformation in solution that is similar to that of PfAldolase, allowing Arg319 to shift and rotate out of the tryptophan-binding site and enable adhesins to bind.

Overall, the greater adhesin-binding site, comprising ∼40 residues, is identical in terms of sequence between the *T. gondii* and *P. falciparum* aldolases, which is not surprising given that the enzymatic role of the protein limits the possible sequences that can be found in this binding site. However, comparison of the structures in terms of the r.m.s.d. of TgAldolase and PfAldolase indicate structural differences that have changed the positioning of multiple residues, which may be owing to crystal artifacts or representative of a mechanism in which aldolases from different organisms can bind adhesin tails of varying sequences. Interestingly, both aldolases must bind adhesin tails of varying sequence, but are not able to change the residues in the binding pocket without the risk of disrupting the enzymatic function. Therefore, in order to accommodate different sequences specific to the species, it is possible that there is flexibility in the binding pocket that allows an induced-fit mechanism of binding.

### 3.3. Future directions   

With an apo TgAldolase crystal structure solved, we hope to proceed with studies to define the binding mode of the microneme-associated protein 2 (MIC2) tail in the TgAldolase active site and to pursue structure-based drug-design studies. Additionally, we would like to use this system to further investigate the binding of *P. falciparum* adhesins by co-crystallization and soaking experiments with TgAldolase and malarial adhesins. There are multiple advantages to using the TgAldolase crystallization system instead of the PfAldolase system to study adhesin binding. In producing TgAldolase crystals using the described methods, we are able to produce numerous crystals. We find that more than 95% of all crystals tested diffract to better than 2 Å resolution, and by using seeding we can obtain these crystals in less than 48 h. The ability to quickly obtain high-quality crystals will aid in studies of PfAldolase, which is more difficult to pursue in crystallization studies. The crystallization of PfAldolase, a protein for which our group and others have determined the apo (Kim *et al.*, 1998 ▶) and peptide-bound states (Bosch *et al.*, 2007 ▶), is less robust, making further studies difficult. Only one in 50 crystals diffracts, and these crystals must be annealed to obtain diffraction to better than 3 Å resolution. The low yield of quality crystals, the time spent screening for ‘collectable’ crystals and the possibility of losing or destroying crystals during the annealing process are reasons causing us to move to the TgAldolase system to further study the interactions of PfAldolase with compounds and adhesin tails. The high reproducibility of the crystallization process in combination with the high sequence identity between TgAldolase and PfAldolase will hopefully make this system a viable tool for studying the interactions that are important in *Plasmodium*.

## Supplementary Material

PDB reference: fructose-1,6-bisphosphate aldolase, 4tu1

Supporting Information.. DOI: 10.1107/S2053230X14017087/dp5074sup1.pdf

## Figures and Tables

**Table 1:** Macromolecule-production information

Source organism	*T. gondii* ME49
DNA source	cDNA
ToxoDB ID	TGME49_236040
ToxoDB sequence†	MSGYGLPISQEVAKELAENARKIAAPGKGILAADESTGTIKKRFDSIGVENTEANRAFYRDLLFSTKGLGQYISGAILFEETLYQKSPSGVPMVDLLKAEGIIPGIKVDKGLETLPLTDDEKATMGLDGLSERCKKYYEAGARFAKWRAVLSIDPAKGKPTNLSITEVAHGLARYAAICQANRLVPIVEPEILTDGSHDITVCAEVTERVLAAVFKALNDHHVLLEGALLKPNMVTHGSDCPKPASHEEIAFYTVRSLKRTVPPALPGVMFLSGGQSEEDASLNLNEMNKMGPHPFQLSFSYGRALQASCLKAWKGVPENKAKAQQVLMERARANGEAQLGKYGGGAGGAAAASSLFEKRYVY
SSGCID Clone ID	TogoA.01236.a.A15.GE33357
Cloning/expression vector	AVA0421 (modified pET-14b with N-terminal His_6_ tag and 3C protease cleavage site)
Expression host	*E. coli* BL21 (DE3)
Complete amino-acid sequence of the construct produced‡	*MA* HHHHHH *MGTLEAQTQGPGSM*ISQEVAKELAENARKIAAPGKGILAADESTGTIKKRFDSIGVENTEANRAFYRDLLFSTKGLGQYISGAILFEETLYQKSPSGVPMVDLLKAEGIIPGIKVDKGLETLPLTDDEKATMGLDGLSERCKKYYEAGARFAKWRAVLSIDPAKGKPTNLSITEVAHGLARYAAICQANRLVPIVEPEILTDGSHDITVCAEVTERVLAAVFKALNDHHVLLEGALLKPNMVTHGSDCPKPASHEEIAFYTVRSLKRTVPPALPGVMFLSGGQSEEDASLNLNEMNKMGPHPFQLSFSYGRALQASCLKAWKGVPENKAKAQQVLMERARANGEAQL
Molecular weight (Da)	38501
Extinction coefficient§ (*M* ^1^cm^1^)/Abs 0.1% (=1gl^1^)	23170

† Sequence based on the note in the ToxoDB database (http://toxodb.org/toxo/) indicating that there is a likely error in the start codon. This construct uses the second start codon in the expected mRNA transcript, which produces a product homologous to other fructose-1,6-bisphosphate aldolases. This construct is 71 amino acids shorter than the gene product listed in the database. The sequence is 363 amino acids in total.

‡ The cloning tag is italicized, with the 6His tag underlined and the 3C protease site underlined. This construct lacks seven amino acids at the N-terminus and 23 amino acids at the C-terminus compared with the sequence deposited in ToxoDB. The sequence is 355 amino acids in total.

§ Calculated using *ProtParam* for the reduced protein sequence.

**Table 2:** Crystallization

Method	Vapor diffusion, sitting drop, seeded
Plate type	Intelli-Plate 24-4 (Art Robbins/Hampton Research)
Temperature (K)	277
Protein concentration (mgml^1^)	20
Buffer composition of protein solution	50m*M* Tris pH 8.0, 50m*M* NaCl, 1m*M* DTT
Composition of reservoir solution	0.1*M* MOPS/HEPESNa pH 7.9, 0.02*M* sodium formate, 0.02*M* ammonium acetate, 0.02*M* trisodium citrate, 0.02*M* sodium potassium L-tartrate, 0.02*M* sodium oxamate, 0.02*M* ammonium acetate, 12.5%(*v*/*v*) glycerol, 25%(*w*/*v*) PEG 4000
Cryocondition	1:3, 100% glycerol:reservoir (final concentration 35%)
Volume and ratio of drop	3l drop, 2:1 (protein:reservoir)
Volume of reservoir (l)	300

**Table 3:** Data collection and processing Values in parentheses are for the outer shell.

Data-set name	14u09_lb05-1
Diffraction source	BL7-1, SSRL
Wavelength ()	1.127
Temperature (K)	100
Detector	ADSC Quantum 315r CCD
Crystal-to-detector distance (mm)	225
Beam size (mm)	0.1 0.1
Rotation range per image ()	0.5
Total rotation range ()	90
Exposure time per image (s)	5
Space group	*P*22_1_2_1_
*a*, *b*, *c* ()	92.26, 134.46, 162.43,
, , ()	90, 90, 90
Mosaicity ()	0.257
Resolution range ()	46.72.0 (2.0712.000)
Total No. of reflections	481063 (47305)
No. of unique reflections	132175 (13009)
Completeness (%)	96.72 (96.22)
Multiplicity	3.6 (3.6)
*I*/(*I*)	15.37 (3.36)
*R*_meas_	0.07236
CC_1/2_	0.999 (0.917)
CC*	1 (0.978)
Overall *B* factor from Wilson plot (^2^)	36.35

**Table 4:** Refinement statistics Values in parentheses are for the outer shell.

Resolution range ()	46.72.0 (2.0712.000)
Completeness (%)	96.72 (96.22)
No. of reflections, working set	132057 (12313)
No. of reflections, test set	6505 (667)
Final *R*_cryst_	0.1940 (0.3331)
Final *R*_free_	0.2442 (0.3773)
No. of non-H atoms	
Total	11268
Protein	10312
Ion	0
Ligand (glycerol)	6
Water	950
R.m.s. deviations	
Bonds ()	0.007
Angles ()	1.02
Average *B* factors (^2^)	
Overall	46.60
Protein	46.80
Ligand (glycerol)	77.90
Water	44.50
Ramachandran plot	
Favored regions (%)	97
Additionally allowed (%)	2.926
Outliers (%)	0.074

## Footnotes

1 Supporting information has been deposited in the IUCr electronic archive (Reference: DP5074).
