# Supplementary material for: Structure of Toxoplasma gondii fructose-1,6-bisphosphate aldolase
Source: Acta Crystallogr F Struct Biol Commun. 2014 Aug 29;70(Pt 9):1186–92. doi: 10.1107/S2053230X14017087 (PMC4157416; doi:10.1107/S2053230X14017087)
Supplement: Supplementary file 1 [file f-70-01186-sup1.pdf]

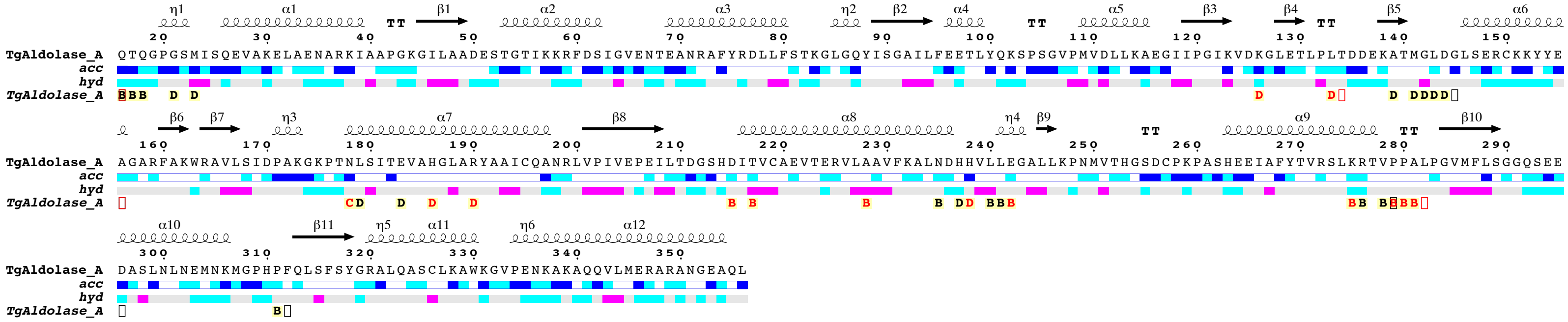

Supplemental Figure 1. ENDscript 2.0 secondary structure mapping of chain A of the TgAldolase structure.

DEFINITIONS:

R-FACTOR
observed = (SUM(ABS(I(h,i)-I(h))))/(SUM(I(h,i)))
expected = expected R-FACTOR derived from Sigma(I)

COMPARED = number of reflections used for calculating R-FACTOR
I/SIGMA = mean of intensity/Sigma(I) of unique reflections (after merging symmetry-related observations)
Sigma(I) = standard deviation of reflection intensity I estimated from sample statistics

R-meas = redundancy independent R-factor (intensities) Diederichs & Karplus (1997), Nature Struct. Biol. 4, 269-275.

CC(1/2) = percentage of correlation between intensities from random half-datasets. Correlation significant at the 0.1% level is marked by an asterisk. Karplus & Diederichs (2012), Science 336, 1030-33
Anomal Corr = percentage of correlation between random half-sets of anomalous intensity differences. Correlation significant at the 0.1% level is marked.
SigAno = mean anomalous difference in units of its estimated standard deviation (|F(+)-F(-)|/Sigma). F(+), F(-) are structure factor estimates obtained from the merged intensity observations in each parity class.
Nano = Number of unique reflections used to calculate Anomal_Corr & SigAno. At least two observations for each (+ and -) parity are required.

SUBSET OF INTENSITY DATA WITH SIGNAL/NOISE >= -3.0 AS FUNCTION OF RESOLUTION

RESOLUTION LIMIT	NUMBER OF REFLECTIONS OBSERVED	UNIQUE	POSSIBLE	COMPLETENESS OF DATA	R-FACTOR observed	R-FACTOR expected	COMPARED	I/SIGMA	R-meas	CC(1/2)	Anomal Corr	SigAno	Nano
50.0000	0	0	16	0.0%	-99.9%	-99.9%	0	-99.00	-99.9%	0.0	0	0.000	0
2.0425	450853	124118	128416	96.7%	5.9%	6.0%	448851	16.17	6.9%	99.9*	-12	0.636	80256
2.0400	1773	479	488	98.2%	40.6%	44.7%	1766	3.40	47.4%	94.2*	0	0.642	321
2.0375	1632	430	440	97.7%	48.6%	53.7%	1631	2.88	56.5%	91.6*	6	0.637	315
2.0350	1715	463	470	98.5%	49.3%	54.3%	1708	2.92	57.5%	91.6*	1	0.635	326
2.0325	1823	488	496	98.4%	43.8%	48.1%	1817	3.28	51.0%	92.1*	-1	0.609	341
2.0300	1667	447	461	97.0%	50.0%	54.7%	1663	2.96	58.3%	94.0*	-1	0.617	309
2.0275	1879	497	513	96.9%	43.9%	45.8%	1873	3.39	51.1%	93.9*	-2	0.676	357
2.0250	1755	472	484	97.5%	44.8%	49.4%	1752	3.18	52.5%	90.8*	-9	0.599	332
2.0225	1681	452	466	97.0%	44.2%	48.3%	1675	3.21	51.6%	92.5*	3	0.638	314
2.0200	1795	482	495	97.4%	50.7%	54.7%	1785	2.92	59.1%	90.6*	-4	0.592	330
2.0175	1785	476	485	98.1%	52.5%	56.3%	1779	2.89	61.1%	90.2*	2	0.622	328
2.0150	1694	445	456	97.6%	50.7%	54.4%	1689	2.90	58.9%	91.5*	-2	0.645	333
2.0125	1857	495	504	98.2%	51.7%	54.9%	1852	2.88	60.3%	90.2*	-6	0.605	341
2.0100	1843	494	503	98.2%	55.8%	59.1%	1836	2.73	65.0%	87.9*	-5	0.621	349
2.0075	1737	458	463	98.9%	49.0%	54.8%	1734	3.03	57.0%	90.8*	-6	0.589	334
2.0050	1876	497	509	97.6%	74.2%	79.6%	1870	2.18	86.2%	81.6*	5	0.629	352
2.0025	1903	513	531	96.6%	50.4%	54.5%	1895	2.97	58.8%	91.5*	-5	0.603	353
2.0000	1804	481	495	97.2%	61.0%	66.4%	1803	2.54	71.1%	90.5*	-6	0.576	340
total	481072	132187	136691	96.7%	6.2%	6.3%	478979	15.36	7.2%	99.9*	-11	0.635	85931

Supplemental Table1. XSCALE statistics of collected data with fine slicing of highest resolution reflections.
